# Predicting genes for orphan metabolic activities using phylogenetic profiles

**DOI:** 10.1186/gb-2006-7-2-r17

**Published:** 2006-02-15

**Authors:** Lifeng Chen, Dennis Vitkup

**Affiliations:** 1 Center for Computational Biology and Bioinformatics and Department of Biomedical Informatics, Columbia University, St Nicholas Avenue, Irving Cancer Research Center, New York, NY 10032, USA

## Abstract

Homology-based methods fail to assign genes to many metabolic activities present in sequenced organisms. To suggest genes for these orphan activities we developed a novel method that efficiently combines local structure of a metabolic network with phylogenetic profiles. We validated our method using known metabolic genes in *Saccharomyces cerevisiae *and *Escherichia coli*. We show that our method should be easily transferable to other organisms, and that it is robust to errors in incomplete metabolic networks.

## Abstract 2

A method that combines local structure of a metabolic network with phylogenetic profiles is described and used to assign genes to orphan metabolic activities in yeast and *Escherichia coli*.

## Background

It is hard to overestimate the potential impact of accurate network reconstruction algorithms on systems biology. Accurate models of biological networks will be essential in diverse areas from genetics of common human diseases to synthetic biology. Current computational methods of metabolic network reconstruction can directly benefit from many decades of experimental biochemical studies [1,2]. Available homology-based annotation methods assign metabolic functions to sequences by establishing sequence similarity to known enzymes. State of the art homology approaches use different types of sequence and structural similarity, such as the overall sequence homology [3-5], presence of conserved functional motifs and blocks [6], specific spatial positions of functional residues [7,8], or a combination of the above [9]. Unfortunately, in spite of the overall success, homology-based methods fail to annotate metabolic genes with poor homology to known enzymes. This has resulted in partially reconstructed metabolic networks, such as for *Escherichia coli *[10] and *Saccharomyces cerevisiae *[11].

The inability to annotate all enzymes using homology-based methods leaves members of metabolic pathways 'missing' [12]. That is, although biochemical evidence may indicate that a certain group of reactions takes place in an organism, we do not know which genes encode the enzymes responsible for the catalyses. It is perhaps natural to call these 'missing' genes orphan metabolic activities, to emphasize the fact that certain metabolic activities are not assigned to any sequences. As suggested by Osterman *et al*. [12], we can classify orphan metabolic activities as 'local' or 'global'. Global orphan activities do not have a single representative sequence in any organism [13]. In contrast, local orphan activities represent reactions for which we do not have a representative sequence in an organism of interest, although one or several sequences catalyzing the reaction may be known in other organisms. The problem of assigning sequences to orphan activities is conceptually conjugate to the problem of assigning activities (functions) to hypothetical sequences. Although progress in solving the former problem will necessarily improve solution of the latter, optimal methods and algorithms for these two problems may be different.

Several non-homology methods have been developed in order to establish functional links between proteins [14,15]. These so-called context-based approaches include gene phylogenetic profiles (measuring co-occurrence of gene pairs across genomes) [16,17], the protein fusion (Rosetta Stone) method (detecting fusion events between genes) [18-20], gene co-expression [21,22], and conserved gene neighborhoods (measuring chromosomal co-localization between genes) [23-25]. It was demonstrated that the functional links generated by the context-based methods recover members of protein complexes, functional modules, molecular pathways and gene-phenotype relationships [26-28].

Previously, Osterman *et al*. [12] illustrated how context-based methods can be successfully used to fill the remaining gaps in the metabolic networks, while Green *et al*. [29] proposed a Bayesian method for identifying missing enzymes using primarily sequence homology and chromosomal proximity information. In contrast to Green, the approach reported here uses exclusively non-homology information. Consequently, our method should be particularly useful when the gene encoding the enzyme catalyzing a particular orphan function has little or no sequence similarity to any known enzymes.

Recently, we used mRNA co-expression data and local structure of a metabolic network to fill metabolic gaps in a partially reconstructed network of *S. cerevisiae *[11]. Using exclusively co-expression information, for 20% of all metabolic reactions it was possible to rank a correct gene within the top 50 out of 5,594 candidate yeast genes.

In this study, we demonstrate that it is possible to significantly improve prediction of sequences responsible for orphan metabolic activities by using gene phylogenetic profiles. Importantly, in contrast to mRNA co-expression data, which are usually available only for several model organisms, phylogenetic profiles can be readily calculated for any sequenced organism. The accuracy of phylogenetic profiles will increase as genomic pipelines reveal more protein sequences. In comparison to previous studies that demonstrated that it is possible to cluster proteins from annotated biochemical pathways using phylogenetic profiles [17,27,30], our goal is significantly more specific in that we want to predict genes responsible for particular orphan activities. By directly taking into account the structure of a partially reconstructed metabolic network (for example, giving more weight to genes closer to a network gap) our method is able to combine the information of a 'known core' of the network with phylogenetic correlations to the remaining gaps. We show that our method is readily applicable to less-studied organisms with partially known metabolic networks.

## Results and discussion

### The main approach

**Figure 1:**
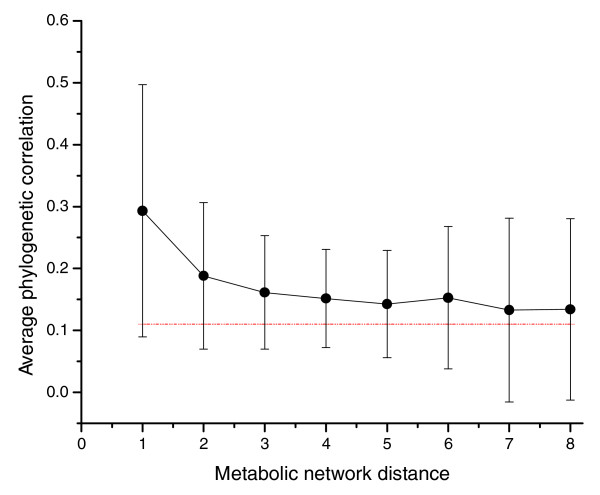
The average phylogenetic correlation between a target gene and all other network genes at a certain metabolic network distance. The standard deviation of the average correlation for all possible network gaps is represented by the error bars. The dashed line shows the background correlation, estimated by the average phylogenetic correlation between any metabolic and non-metabolic genes. The average phylogenetic correlation between two genes decreases monotonically with their separation in the network.

As was demonstrated by us previously [31,32], the closer genes are in a metabolic network the more similar are the genes' evolutionary histories. It is important to know whether this relationship is strong enough to determine the exact network location of a hypothetical gene. The established distance metrics (see Materials and methods) allows us to quantify the relationship between the gene distance in the network and the average gene co-evolution (Figure 1). In Figure 1 we show Pearson's correlations of phylogenetic profiles between a target gene and all other network genes separated from the target by distances one, two, three, and so on. The background correlation (0.11) was estimated by averaging correlation coefficients between all non-metabolic and metabolic genes. The average correlation between metabolic genes decreases monotonically with their separation in the metabolic network, ranging between 0.29 for metabolic distance 1 and 0.13 for metabolic distance 8. This relationship suggests that we can use gene phylogenetic profiles and their location in the metabolic network to predict sequences for orphan activities.

The idea behind our method is similar to that used by us previously in the context of mRNA co-expression networks [31]. We used a heuristic cost function to determine how a test gene 'fits' into a network gap. The 'fit' of a test gene in a network gap is determined by its phylogenetic correlations with network genes close to the gap. The parameters of the cost function were optimized to achieve the best predictive ability by minimizing the log sum of the ranks for all correct metabolic enzymes. Several functional forms of the cost function were tested (see Equations 1 to 3 below).

Equation 1 represents a cost function similar to the one used previously [31], where *x *is the candidate gene, *n *is a gene from the network neighborhood of the gap, *c*(*x*, *n*) is the phylogenetic correlation between genes *x *and *n*,  is the vector of layer weights, and *p1 *is the power factor for the phylogenetic correlations. The summation in Equation 1 is, first, over all genes in a given layer *N*_*i *_around the gap and, second, over all layers up to the layer *R*. Only three layers around the network gaps were used in all calculations in the paper. |*N*| is the total number of genes in all three layers.



Equation 2 represents a cost function that takes into account the specificity of connections established by metabolites. The idea behind the connection specificity is the following: if a metabolite participates in establishing few connections (that is, the metabolite participates in a small number of reactions), the corresponding connections are given more weight in the cost function compared to connections established by widely used metabolites. The connection specificity was taken into account by an additional weight parameter  (*g*, *n*), determined by an inverse power function of the total number of connections established by the metabolite linking the gap gene *g *and its neighboring gene *n*. If more than one metabolite establishes the connection between *g *and *n*, the most specific one (the metabolite with the fewest connections) was used.



Equation 3 represents an exponential cost function, which is used to increase the sensitivity to differences between phylogenetic correlations. A set of new parameters (*β*_*i*_) was introduced to account for different weighting of the exponent in different layers.



We found that the functions with connection specificity adjustment (Equations 2 and 3) significantly outperform the function without specificity adjustment (Equation 1). However, we found no difference in predictive power between Equation 2 and 3 (Additional data file 4). In the text below, unless otherwise specified, we present results obtained using Equation 2.

### Self-consistent test and parameter optimization

**Figure 2:**
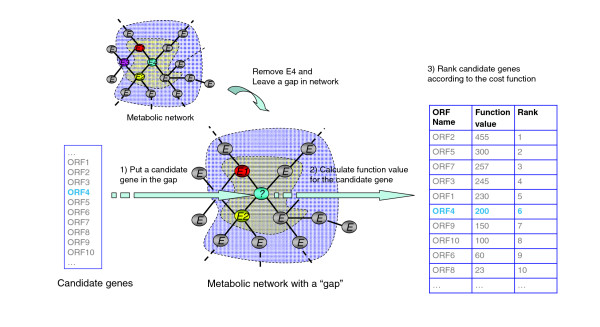
'Fit' test of a candidate gene in a network gap. We use a self-consistent test in which a known gene E4 is removed from the network, leaving a gap in its place. We then: 1, put candidate genes in the gap one by one; 2, determine the function value for every candidate gene (Equations 1 to 3); and 3, rank all candidate genes based on their function values. In the figure we show an example when the correct gene E4 was ranked as number 6.

To optimize the cost function parameters and assess the performance of our method we carried out a self-consistent test illustrated in Figure 2. The test consists of: removing a known gene from its position in the network (leading to a network gap); adding the gene to a collection of 6,093 non-metabolic yeast genes; and ranking all candidate genes in terms of their 'fit' in the network gap according to the cost function. As the correct gene occupying the gap is known, we can accurately measure the performance of the method based on the obtained ranking. The overall performance of the method was quantified by calculating the fraction of correct genes that are ranked as the top, within the top 10 and within the top 50 out of all non-metabolic yeast genes. These performance measures are directly related to the main goal of our method: to suggest candidates for orphan activities to be tested experimentally. Even if our method is not always able to rank the correct gene as the top candidate, it may be useful, for example, to rank it within the top 10 candidates. These top 10 candidates can then be tested experimentally to find out the exact gene responsible for the orphan activity.

The optimal values for the cost function parameters were determined by minimizing the log sum of the ranks of all known metabolic enzymes in their correct network positions (see Materials and methods). Two types of parameter optimization algorithm were used: a deterministic Nelder-Mead simplex algorithm [33] and a stochastic global optimization by simulated annealing (SA) [34]. The best performance was obtained from the SA optimizations and is reported below.

**Figure 3:**
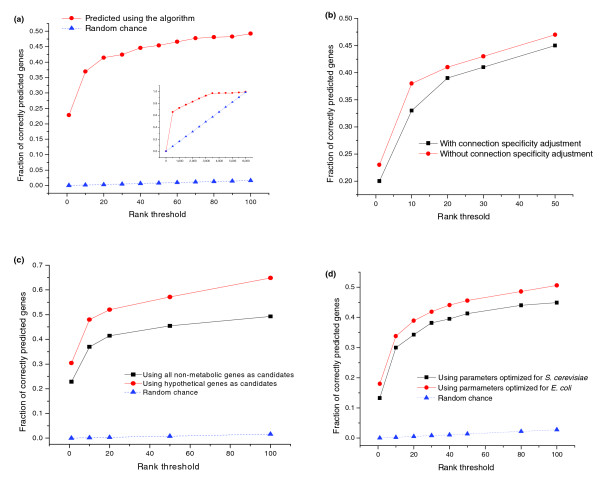
Enzyme predictions based on phylogenetic profiles. **(a) **The cumulative fraction of correctly predicted genes as a function of rank among all non-metabolic genes. All 6,093 non-metabolic yeast genes plus a known correct gene were ranked using Equation 2. The cumulative distribution is shown for ranks from 1 to 100; the inset shows the same distribution for all ranks. **(b) **The effect of connection specificity adjustment. Only highly ranked genes (1 to 50) are shown. **(c) **Comparison of the performance with all non-metabolic genes as candidates to that with only hypothetical genes as candidates for an orphan activity. **(d) **Predictions for the *E. coli *metabolic network. The cost function with the parameters optimized for the yeast network showed comparable performance to the cost function with the parameters specifically optimized for the *E. coli *network.

The optimized prediction algorithm identifies 22.8%, 37.3% and 46.2% of the correct genes as the top candidates, within the top 10 candidates, and within the top 50 candidates out of 6,094 genes, respectively (Figure 3a). In comparison, under random ranking, the fraction of correct genes as the top candidate, within the top 10 candidates, and within the top 50 candidates is only 0.016%, 0.16% and 0.8%, respectively. For Equation 2, optimal performance was observed with the correlation power *p1 *= 1.81 (95% confidence interval (CI): 1.40-2.21) and the connection specificity power *p2 *= 0.79 (95% CI: 0.68-0.90). As the ratio of the number of the cost function adjustable parameters to observations is around 1:100, our method does not suffer from overfitting. We achieved almost identical prediction accuracies using the training and test sets in ten-fold cross-validation (Additional data file 5).

The functional information present in the currently available phylogenetic profiles allows us to significantly improve the performance in comparison to a similar method based on gene co-expression. Using mRNA co-expression, we predicted 4.1%, 12.7% and 23.8% of the correct enzyme-encoding genes to be top ranked, within the top 10, and within the top 50, respectively [31]. The improved performance reflects larger coverage of the available phylogenetic profiles, which can be calculated for many sequences in various genomes; in contrast, mRNA co-expression data are mostly available for model organisms and genes with significant mRNA expression changes. Another important improvement of the current approach is the use of the connection specificity adjustment. The specificity adjusted cost functions (Equations 2 and 3) predict 5% to 18% more correct genes within the top ranks compared to functions without specificity adjustment (Equation 1; Figure 3b).

It is interesting to investigate the relative contribution of different layers around a network gap to the cost function. As only the relative difference in layer weights impact the algorithm performance, the weight of the first layer was always set to 1. The best performance of the algorithm based on Equation 2 was achieved with the following weights for the second and third layers around the gap: *w2 *= 0.0085 (95% CI: 0.0051-0.0120) and *w3 *= 0.0024 (95% CI: 0.0011-0.0037). Smaller values for the weights *w2 *and *w3 *indicate that the phylogenetic correlations at the distances 2 and 3 from the gap are not as informative as the correlations of the first layer neighbors. But, as there are 5 and 13 times more genes in the second and third layers, respectively, their contribution to the cost function values is around 5% to 10% for the highly ranked genes and more than 10% for enzymes ranked between 200 and 600. As we show below, the contribution of the second and third layers roughly doubles for predictions on partially known networks.

### Performance based on phylogenetic profiles generated using COG

As described in Materials and methods, BLAST searches were used in this work to calculate phylogenetic profiles. In contrast, a number of previous studies [27,35] relied on the Cluster of Orthologous Groups (COG) database [36] to obtain phylogenetic profiles. We investigated the performance of our algorithm on COG-based phylogenetic profiles. Using the same algorithm and the COG-based profiles, we predicted 34.1%, 56.2% and 69.0% of the correct yeast metabolic genes to be the top ranked, within the top 10 and within the top 50, respectively. This indicates an improvement of about 50% over the results based on the BLAST searches; however, this result is unlikely to indicate superior performance. First, the current coverage of the COG database is significantly biased towards genes encoding known metabolic enzymes. For example, 72% (443 out of 615) of known metabolic genes have COG profiles while only 19% (1,148 out of 6,093) of non-metabolic genes have COG profiles. This bias leads to a significant overestimation of the 'real-world' performance of the COG-based profiles. Second, the COG database has a very limited set of hypothetical proteins, making it impractical to predict hypothetical genes responsible for orphan activities using COG.

### Performance using hypotheticals as candidate genes

In practice, it is logical to test only hypothetical genes for orphan metabolic activities in a given organism. To simulate this for the yeast metabolic network, we repeated our self-consistent test procedure using only hypothetical yeast genes as gap candidates. We identified 1,514 hypothetical yeast open reading frames (ORFs) for this analysis. As the number of hypothetical genes is smaller than the total number of genes (usually 30% to 70% smaller), the performance of our method should improve. Indeed, testing only hypothetical genes improved the algorithm performance: 30.4%, 48.0% and 57.1% correct enzymes were ranked as the top 1, within the top 10 and within the top 50 among all candidate sequences, respectively (Figure 3c). We note that the observed 25% improvement in performance is not due to a better discrimination against hypothetical genes. Similar improvement was observed when a candidate set of 1,514 randomly selected genes with known functions was used (Additional data file 6).

### Performance on the *E. coli *metabolic network

To understand the transferability of our approach to other organisms, we repeated our analysis using the *E. coli *metabolic network. The same procedures were used to construct the metabolic network for *E. coli *(see Materials and methods). First, the optimal parameters obtained for the *S. cerevisiae *metabolic network, without further modifications, were applied to rank *E. coli *metabolic genes. As a result, the algorithm predicts 13.3%, 30.0%, and 41.3.% of known *E. coli *metabolic genes to be top ranked, within the top 10 and within the top 50, respectively, out of 3,578 non-metabolic *E. coli *genes. Second, the simulated annealing optimization was performed to optimize the cost function specifically for the *E. coli *network. Based on the optimized parameters slightly better results were obtained: 18.0%, 33.8%, and 45.6% of the correct genes were ranked as the top candidate, within the top 10, and within the top 50, respectively (Figure 3d). The optimal *E. coli *parameters for the cost function are generally similar to the optimal parameters for the *S. cerevisiae *metabolic network. This suggests that parameters obtained on several model organisms can be directly used for predictions in other organisms, although an organism-specific optimization will slightly improve the algorithm performance.

### Performance based on genes without independent homology information

Our prediction method is designed primarily for enzymatic activities without good homology information. Above, we validated the approach using all known metabolic enzymes from *E. coli *and *S. cerevisiae*. In addition, it is interesting to identify a set of enzymes for which independent homology information is not available (that is, the biochemical experiments have been conducted only in *E. coli*, for example) and test the performance on this subset.

We obtained a subset of *E. coli *enzymatic EC numbers without representative sequences in other organisms. The subset, identified using the SWISS-PROT database [37], includes EC numbers with representative sequences exclusively from *E. coli*. We also included EC numbers with representative sequences in the TrEMBL database (a computer-annotated complement to the SWISS-PROT), but only if these were computationally annotated from *E. coli *sequences and, consequently, cannot provide independent homology information. Each identified EC number was then manually checked.

The identified subset consists of 25 enzymes and is listed in Table 1. The performance of our method on the subset was comparable to the performance observed for the set of all *E. coli *enzymes: 16.0%, 24.0% and 44.0% of the correct enzymes were ranked as the top, within the top 10, and within the top 50, respectively, among all *E. coli *candidate genes. Consequently, the algorithm is effective for sequences that are likely to be missed by homology-based methods.

### Importance of the neighborhood

**Figure 4:**
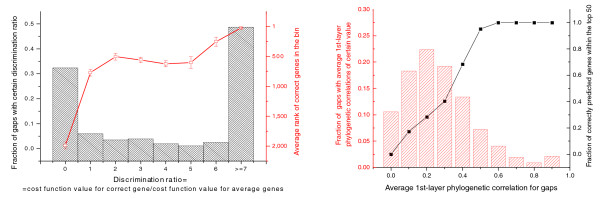
Importance of metabolic neighborhood for the predictive power of the algorithm. **(a) **Informative and non-informative gaps. About one-third of the gaps did not allow any discrimination between the correct and average genes (represented by bin 0 in the figure), that is, the function value of the correct gene is equal to or smaller than the function value for average genes determined by Equation 2. The red line shows the average rank of correct genes represented in each bin. Genes filling gaps with higher discrimination ratios are ranked higher by the algorithm. **(b) **The relationship between the rank of a correct enzyme in a gap and the average correlation of first layer genes around the gap. A metabolic gene for a gap with a high average first layer correlation (>0.5) is usually highly ranked by the prediction algorithm (black line) but the fraction of such gaps is small (red bins).

The performance of our algorithm for a specific network gap should crucially depend on the available evolutionary information for network genes located around the gap. As we optimized our algorithm we found that for about one-third of all gaps the algorithm performance is no better than random. To investigate this further, we calculated the discrimination ratio of the cost function value for the correct gene and the average for all non-metabolic genes. The distribution of the discrimination ratios for all possible gaps in the metabolic network is shown in Figure 4a. Confirming our expectation, about one-third of all gaps did not allow any discrimination between the correct and average genes (bin 0 in Figure 4a represents gaps with discrimination ratios less than 1). On the other hand, about 50% of the gaps have discrimination ratios equal or greater than 7 (bin >= 7 in Figure 4a). For comparison, the average rank of the correct genes for the gaps in bin 0 is only 1,989, while it is 26 for the gaps in bin >= 7.

We found that an important feature that separates the informative and non-informative gaps is the availability of accurate phylogenetic correlations for the neighborhood genes around the gaps. Clearly, if accurate phylogenetic correlations cannot be calculated - because, for example, the corresponding genes exist only in several related genomes - the cost function will not be able to discriminate between correct and incorrect genes. Figure 4b illustrates this point by showing the relationship between the average phylogenetic correlation between the first layer genes and the fraction of well-predicted gaps. For gaps with a first layer correlation of at least 0.5, 95% of the correct genes are ranked within the top 50. In contrast, less than 20% of the correct genes are ranked within the top 50 if the average first layer correlation is below 0.1. In practice, the discrimination ratio can be used to estimate the predictive ability of different gaps.

### Performance based on a partially known networks

**Figure 5:**
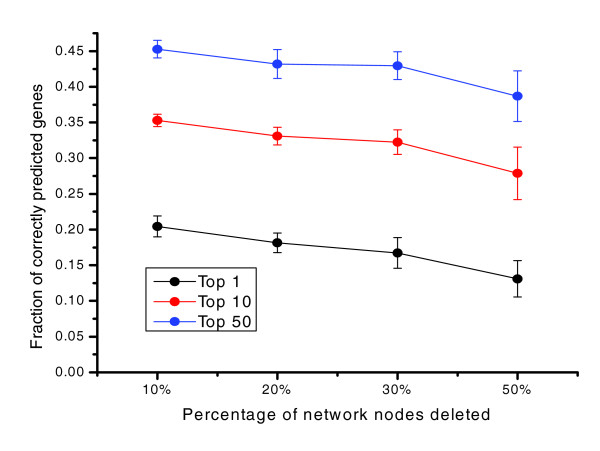
The algorithm performance using an incomplete metabolic network. We show the algorithm performance for yeast networks with a certain fraction of genes randomly deleted. The performance decrease is gradual as up to 50% of the network nodes are deleted. For example, when half of the network is deleted, we can still predict more than 33% of the correct metabolic genes within the top 50 among all candidate genes, compared to 0.8% by random chance.

Currently available metabolic networks are significantly incomplete. As our algorithm directly relies on the network structure, it is important to understand that the algorithm performance depends on the network completeness. To investigate this we deliberately removed a certain fraction of known genes from the yeast network and retrained our algorithm on the incomplete network. We tried two approaches to simulate incomplete networks. First, we completely deleted a fraction of genes from the network and removed all connections to the deleted genes. Second, we effectively converted a fraction of the metabolic network into orphan activities. In this case the connections established by the orphan activities are preserved, but the genes responsible for these activities are converted into orphan activities. These two deletion approaches gave similar results and we report here only the effects of complete gene deletions. As Figure 5 demonstrates, the performance of our method decreases only gradually when increasing fractions of network genes are deleted. Even when as many as 50% of the network genes are deleted, the algorithm still performs reasonably well, predicting 13.7% as the top candidate (95% CI: 10.5-15.6%), 27.9% to be within the top 10 (95% CI: 24.2-31.5%), and 33.1% within the top 50 (95% CI: 29.2-37.1%). Interestingly, when a high percentage (20% to 50%) of the network was deleted, the relative cost function contributions from genes of the second and third layers around gaps increased approximately twice. This suggests that, for an incomplete network, the second and third layers play a larger role in 'focusing' a correct gene towards the corresponding gap.

The relative insensitivity of our method to the network completeness suggests that the algorithm based on phylogenetic profiles will be useful not only for metabolic networks of model organisms, such as *S. cerevisiae *and *E. coli*, but also for networks of less studied organisms.

### Predictions for orphan activities in *S. cerevisiae *and *E. coli*

As the metabolic networks of *E. coli *and *S. cerevisiae *are relatively well studied, it is likely that the developed algorithm will be most useful in less studied species with a larger fraction of orphan metabolic activities. Nevertheless, we investigated in detail several predictions for orphan activities in the *E. coli *and *S. cerevisiae *networks.

Although considered as gaps in the originally reconstructed *E. coli *[10] and *S. cerevisiae *networks [11], a number of orphan activities have been recently identified. For example, the yeast enzyme 5-formyltetrahydrofolate cyclo-ligase (EC 6.3.3.2) appears as a gap in the network model by Forster *et al*. [11]. However, the gene responsible for this activity, YER183C/FAU1, has been cloned and characterized by Holmes and Appling [38]. This gene is present in the updated model by Duarte *et al*. [39]. In the *E. coli *iJR904 model, the arabinose-5-phosphate isomerase (API, EC 5.3.1.13) is listed as an orphan activity. However, the *yrbH*/b3197 gene has been recently characterized as encoding the enzyme responsible for this metabolic reaction [40]. Significantly, without any sequence homology information, our algorithm was able to rank the *S. cerevisiae *FAU1 gene and the *E. coli yrbH *gene as the number 10 and number 1 candidate, respectively, for their corresponding enzymatic activities. More examples for recently identified orphan activities and predictions can be found in Additional file 9.

Several orphan activities in *S. cerevisiae *and *E. coli *remain unassigned to any gene. We found several interesting predictions for the NAD+ dependent succinate-semialdehyde dehydrogenase (EC 1.2.1.24) in *E. coli*. *E. coli *seems to possess two different types of succinate semialdehyde dehydrogenases [41]: one is NAD(P)+ dependent and is encoded by the b2661/*gabD *gene (EC 1.2.1.16); the other is specific for NAD+ only (EC 1.2.1.24). One *E. coli *gene, b1525/*yneI*, was predicted as the top candidate for this orphan activity. We believe *yneI *is a good candidate for the orphan activity because of the following additional functional clues. It has 32% sequence identity (E-value 5*10^-61^) to the other *E. coli *succinate semialdehyde dehydrogenase encoded by *gabD *and 30% sequence identity to the human enzyme ALDH5A1 (EC 1.2.1.24, E-value 7*10^-59^). In addition, *yneI *is adjacent on the bacterial chromosome to the gene *yneH*/*glsA2*/b3512, which encodes glutaminase 2 (EC 3.5.1.2). The gene *yneH *is involved in the same glutamate metabolism pathway as EC 1.2.1.24. The closeness of *yneI *and *yneH *on the chromosome suggests that they are involved in related functions.

## Conclusion

We demonstrate in this work that genes encoding orphan metabolic activities can be effectively identified by integrating phylogenetic profiles with a partially known network. The reported approach is significantly more accurate in comparison to a similar method based on mRNA co-expression [31]. We are able to predict five times more correct genes as the top candidates and two times more within the top 50 candidates out of about 6,000 unrelated yeast genes. It is likely that the improvement in performance reflects larger functional coverage of the available phylogenetic profiles over mRNA co-expression data. Indeed, the performances of the algorithms based on mRNA co-expression and phylogenetic profiles are similar when only well-perturbed network neighborhoods, the neighborhoods with large changes in gene expression, are considered.

The larger functional coverage of phylogenetic profiles allows our approach to be extended to organisms with no or little expression data. As we demonstrate, the optimized parameters are likely to be directly transferable between organisms. Importantly, the incompleteness of the currently available metabolic networks is not a major hindrance to the application of our algorithm.

The performance of our algorithm significantly improves if the specificity of the connections established by different metabolites is taken into consideration. To account for the connection specificity, the algorithm assigns smaller cost function weights to connections established by widely used (that is, non-specific) metabolites. Similar specificity corrections should be useful for calculations based on other context-based descriptors, such as mRNA expression.

**Figure 6:**
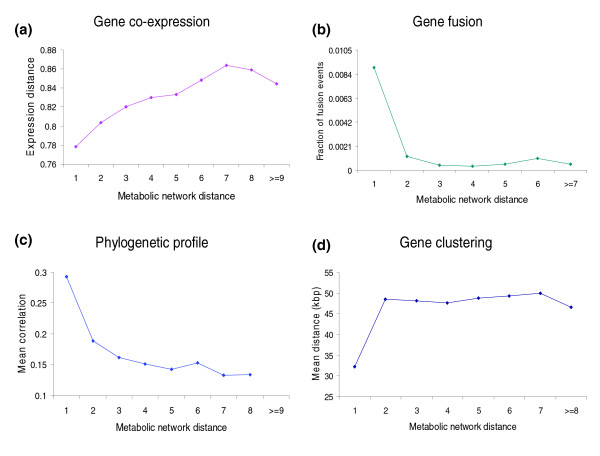
Context-based associations versus the metabolic network distance for the yeast metabolic network. **(a) **mRNA expression distance. The expression distance is calculated as 1-|correlation|, where correlation is the Spearman's rank correlation between genes' mRNA expression. Close neighbors in the metabolic network have similar expression profiles. **(b) **Gene fusion events (Rosetta Stone). The fraction of proteins involved in gene fusion events. The adjacent genes in the network are much more likely to form a Rosetta Stone protein. **(c) **Phylogenetic profiles. Pearson's correlations between phylogenetic profiles for genes close in the network are more likely to be similar. **(d) **Chromosomal distance between genes. The mean physical distances (in kilobase pairs (kbp)) between ORFs are shown. The adjacent genes in the network are significantly closer to each other on yeast chromosomes.

Ultimately, to achieve maximal performance it will be necessary to combine various sequence-based and context-based descriptors. In Figure 6 we show how different context-based associations change as a function of the network distance between the metabolic genes. Four different context-based associations are shown: gene co-expression, gene fusions (Rosetta Stone), phylogenetic profiles, and chromosomal gene clustering (similar relationships for *E. coli *are shown in Additional data file 7). The figures demonstrate that different context-based associations can contribute to 'focusing' a hypothetical gene to its proper location in the network. We are currently building a combined method (P. Kharchenko, L.C., Y. Freund, D.V., G.M. Church, unpublished data) that will integrate different associations in order to predict genes responsible for orphan metabolic activities. We also plan to apply similar gap-filling methods to other cellular networks.

## Materials and methods

### Construction of metabolic networks

**Figure 7:**
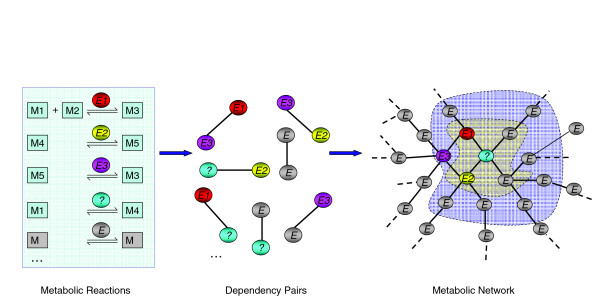
Construction of a network from a list of metabolic reactions. The direct connections are established between the dependency pairs: gene pairs sharing metabolites (M) as reactants or products. An orphan activity (metabolic network gap) is marked by a question mark and surrounded by known metabolic genes. The first and second network layers around the gap are colored yellow and blue, respectively. E, enzyme.

We used the manually curated metabolic reaction set of Forster *et al*. [11] to construct the *S. cerevisiae *metabolic network. The reaction set consists of 1,172 metabolic reactions. The method to build a metabolic network from a reaction set has been described elsewhere [31,32] and is illustrated in Figure 7. The nodes of the network correspond to metabolic genes, and the edges correspond to the connections established by metabolic reactions (Figure 7). Two metabolic genes are connected if the corresponding enzymes share a common metabolite among their reactants or products. By calculating the shortest path between any two metabolic genes we established the network distance metrics. Orphan metabolic activities appear in the network as gaps (Figure 7). We refer to 'first layer neighbors' (yellow in Figure 7) of a target gene to describe the collection of genes with distance one to the target gene, 'second layer neighbors' (blue in Figure 7) to describe the genes with distance two, and so on.

While any metabolite can be used to establish connections between metabolic genes, common metabolites and cofactors, such as ATP, water or hydrogen, are not likely to connect genes with similar metabolic functions. Indeed, the performance of our algorithm on the network in which all connections were present was significantly worse than on the network in which highly connected metabolites were excluded [31]. In order to determine an exclusion threshold, we gradually removed the most highly connected metabolites while monitoring the overall performances of the algorithm. We found that the best performance was achieved when the 15 most highly connected metabolites were excluded from the network reconstruction. Exclusion of more than the 15 most connected metabolites increases prediction accuracy by a slight margin, although the coverage of metabolic genes in the network is reduced significantly. For instance, 20% and 50% metabolic genes lost all their network connections when 120 and 240 most frequent metabolites were excluded, respectively, while the network retains more than 99% of all metabolic genes when only the 15 most frequent metabolites were excluded. The results presented in this paper are thus based on the metabolic network constructed without these 15 most frequent metabolites: ATP, ADP, AMP, CO2, CoA, glutamate, H, NAD, NADH, NADP, NADPH, NH3, GLC, orthophosphate and pyrophosphate.

The reconstructed yeast network contains 615 known metabolic genes and 230 orphan activities. On average, a metabolic gene has 15.8, 76.2 and 200.0 neighbors on its first, second and third layers in the neighborhood, respectively. The average distance between a pair of metabolic genes in the yeast network (network radius) is 3.48. In a similar manner as for *S. cerevisiae*, we constructed the metabolic network for *E. coli *from the iJR904 model by Reed *et al*. [10]. Again, the 15 most frequent metabolites were excluded. The *E. coli *network contains 613 known metabolic enzymes and 136 orphan activities with a network radius of 3.81.

### Phylogenetic profile measures

#### Binary phylogenetic profiles

We constructed phylogenetic profiles for all 6,708 *S. cerevisiae *and 4,199 *E. coli *ORFs using automated BLAST searches against a collection of 70 prokaryotic and eukaryotic genomes (Additional data file 1). Our collection of genomes is similar to the one used by Bowers *et al*. [26]. We deliberately filtered evolutionarily similar genomes. To calculate phylogenetic profile correlations between genes we used a 70-dimensional binary vector representing presence or absence of homologs of a target yeast or *E. coli *gene in query genomes. The Pearson's correlation between the profile vectors (31) was calculated using Equation 4:



where *N *is the total number of the lineages considered. For genes *X *and *Y*, *x *is the number of times *X *occurs in the *N *lineages, *y *is the number of times *Y *occurs in the *N *lineages, and *z *is the number of times *X *and *Y *occur together.

Naturally, our calculations of phylogenetic profiles rely on the BLAST E-value threshold used for considering protein homology of target genes. In the study by Bower *et al*. an E-value of 10^-10 ^was used [26]. We tried different E-value cutoffs (10^-2 ^to 10^-12^) looking for the best algorithm performance. We found that an E-value of 10^-3 ^gave significantly better results in comparison with either more (10^-10^) or less stringent (10^-2^) thresholds; 3 and 5 times better, respectively. In this report, unless otherwise specified, the binary phylogenetic profile correlations were calculated using E = 10^-3 ^as the homology threshold.

#### Normalized phylogenetic profiles and mutual information

Date *et al*. [42] introduced the use of normalized phylogenetic profiles to infer functional associations. Instead of using a predetermined E-value threshold to determine the presence of a homolog for a protein *i *in a genome *j*, they proposed using the value -1/logE_*ij*_, where E_*ij *_is the BLAST E-value of the top-scoring sequence alignment hit for the target protein *i *in the query genome *j*. In this way different degrees of sequence divergence are captured without a predefined cutoff. We calculated the Pearson's correlation coefficients between the normalized phylogenetic profiles for all *S. cerevisiae *and *E. coli *genes.

The study by Wu *et al*. [30], together with the study by Date *et al*. [42], also suggested using mutual information (MI) to assess protein functional association. We calculated MI according to Equation 5:

*MI*(*A*, *B*) = *H*(*A*) + *H*(*B*) - *H*(*A*, *B*)     (5)

where *H*(*A*) = -∑*p*(*a*)*lnp*(*a*) represents the marginal entropy of the probability distribution *p*(*a*) of gene *A *of occurring among all query genomes and *H*(*A*, *B*) = -∑*p*(*a*, *b*)*lnp*(*a*, *b*) represents the relative entropy of the joint probability distribution *p*(*a*, *b*) of the genes *A *and *B *occurring across all the query genomes used in this study. Two sets of MI, based on the binary and normalized phylogenetic profiles described above, were generated and used in our prediction.

We tested the effect of normalized phylogenetic profiles as well as mutual information in our algorithm but did not detect any significant improvements compared to binary profiles (Additional data file 8). Since the procedure of generating binary phylogenetic profiles is more straightforward, in this report, unless otherwise specified, we use the correlations generated using binary phylogenetic profiles (E = 10^-3^).

#### COG-based phylogenetic profile

In addition to using BLAST searches to generate phylogenetic profiles, we also utilize the COG database [36] as the source of orthology information to create phylogenetic profiles. We used the January 2005 version of the COG database consisting of 44 genomes. We consider that an ortholog of a target ORF exists in a query genome if a sequence from that genome co-occurs in the same COG as the target gene. Based on the COG orthology information, a binary phylogenetic profile string was calculated for each gene and pair-wise correlations were calculated using Equation 4.

#### Cost function optimization

Two methods were used to optimize the parameters of the cost functions. First, following our previous analysis [31], the layer weights were optimized using the Nelder-Mead simplex algorithm [33]. The simplex optimization usually took 6 to 8 hours to converge on a Dell PowerEdge 1750 with Dual CPUs at 2.8 GHz and 2 GB DDR SDRAM memory. We usually carried out the simplex optimizations starting from many (10 to 15) randomly chosen starting points to check the sensitivity towards initial conditions. Second, because the simplex algorithm is deterministic and may miss a global parameter minimum, we also used a global SA algorithm [34]. We used the SA algorithm to optimize all parameters used in the cost functions, including the layer weights and the power factors for both phylogenetic correlations and connection specificities. Several annealing schedules were tried. Naturally, the SA algorithm took much longer (usually >20 hours on the same machine) to converge.

Using the SA optimization we obtained lower average ranks for correct metabolic genes and thus better overall performance. For this reason, the results reported in the paper are based on the SA optimization. However, we want to point out that these two algorithms (SA and the simplex) have comparable performance on highly ranked genes (ranked 1 to 100; Additional data file 3). Since our ultimate goal is to generate a list of highly probable candidates for orphan activities to be tested experimentally, the number of candidate genes for each gap should probably not exceed 50 to 100. Thus, the simplex algorithm, although not optimal, is probably sufficient for this purpose.

#### Ten-fold cross-validation

We carried out a ten-fold cross-validation to estimate the accuracy of our method and generalization errors. The set of the known enzymes was randomly split into ten groups. One such group of enzymes was left out each time and designated as the test group. We then trained our method on the remaining 90% of the enzymes and used the obtained parameters to evaluate the performance on the test group.

#### Performance on partially known network

To evaluate the performance on incomplete metabolic networks, we deliberately deleted up to 50% of the enzyme nodes in the *S. cerevisiae *metabolic network. The deleted nodes were added to the candidate gene set, and the performance of the algorithm was evaluated using the incomplete network. This experiment was repeated ten times and the results averaged.

## Additional data files

The following additional data are available with the online version of this paper. Additional data file 1 is a table showing the genomes used in this study to generate phylogenetic profiles. Additional data file 2 is a table showing the effect of connection specificity adjustment. Additional data file 3 is a figure comparing the performance of the simplex and simulated annealing algorithms. Additional data file 4 is a figure comparing the predictions based on Equations 2 and 3. Additional data file 5 is a figure showing 10-fold cross-validation of the algorithm. Additional data file 6 is a figure comparing the predictions based on all yeast non-metabolic genes as the candidate gene set, all hypothetical genes or a randomly selected subset of yeast non-metabolic genes. Additional data file 7 is a figure showing context-based association as a function of metabolic network distance in *E. coli*. Additional data file 8 compares predictions based on normalized gene phylogenetic profiles, mutual information, and the method reported in the paper. Additional data file 9 is a dataset of sample predictions for *E. coli *and *S. cerevisiae *orphan activities.

## Supplementary Material

Additional File 1 Genomes used in this study to generate phylogenetic profiles. Click here for file

Additional File 2 The effect of connection specificity adjustment. Click here for file

Additional File 3 Comparison of the performance of the simplex and simulated annealing algorithms. Click here for file

Additional File 4 Comparison of the predictions based on Equations 2 and 3. Click here for file

Additional File 5 Ten-fold cross-validation of the algorithm. Click here for file

Additional File 6 Comparison of the predictions based on all yeast non-metabolic genes as the candidate gene set, all hypothetical genes or a randomly selected subset of yeast non-metabolic genes. Click here for file

Additional File 7 Context-based association as a function of metabolic network distance in *E. coli*. Click here for file

Additional File 8 Comparison of predictions based on normalized gene phylogenetic profiles, mutual information, and the method reported in the paper. Click here for file

Additional File 9 Dataset of sample predictions for *E. coli *and *S. cerevisiae *orphan activities. Click here for file

## Figures and Tables

**Table 1:** Performance of our method with *Escherichia coli *orphan activities without independent sequence homology information

EC number	Description	Responsible gene	Rank
1.14.11.17	Taurine dioxygenase	b0368/tauD	1,143
1.1.1.251	Fructose 6-phosphate aldolase	b0825/fsa	1
1.1.1.264	L-idonate 5-dehydrogenase	b4267/idnD	44.5
1.2.1.22	Lactaldehyde dehydrogenase	b0356/adhC	1
1.2.1.22	Lactaldehyde dehydrogenase	b1241/adhE	18
1.2.1.22	Lactaldehyde dehydrogenase	b3588/aldB	208
1.2.1.22	Lactaldehyde dehydrogenase	b1415/aldA	654
1.2.2.2	Pyruvate oxidase	b0871/poxB	1,451
1.2.1.39	Phenylacetaldehyde dehydrogenase	b1385/feaB	1
1.1.1.57	Mannonate oxidoreductase	b4323/uxuB	71.5
1.1.1.77	Lacaldehyde reductase	b2799/fucO	10
2.7.1.130	Tetraacyldisaccharide 4'kinase	b0915/lpxK	1,507
2.6.1.66	Valine-pyruvate aminotransferase	b3572/avtA	70.5
2.7.1.58	2-Dehydro-3-deoxygalactonokinase	b3693/dgoK	68
2.7.1.73	Insosine kinase	b0477/gsk	1,041
3.2.2.4	AMP nucleosidase	b1982/amn	69
2.7.7.58	2,3-Dihydroxybenzoate adenylate synthase	b0594/entE	30
4.1.2.20	5-Dehydro-4-deoxyglucarate aldolase	b3126/garL	2,057.5
4.1.1.41	Methylmalonyl-CoA decarboxylase	b2919/ygfG	889
4.1.1.47	Glyoxalate carboligase	b0507/gcl	1
4.2.1.42	Galactarate dehydratase	b3128/garD	757
4.2.1.6	galactonate dehydratase	b3692/dgoA	1,841.5
4.2.1.7	Altronate hydrolase	b3091/uxaA	25
5.3.1.22	Hydroxypyruvate isomerase	b0508/hyi	33
6.2.1.30	Phenylacetate-CoA ligase	b1398/paaK	9

The subset of orphan activities, identified using the SWISS-PROT database [37], includes EC numbers with representative sequences exclusively from *E. coli*. We also included EC numbers with representative sequences in the TrEMBL database, but only if these were computationally annotated from *E. coli *sequences.
