# Supplementary material for: Predicting genes for orphan metabolic activities using phylogenetic profiles
Source: Genome Biol. 2006 Feb 15;7(2):R17. doi: 10.1186/gb-2006-7-2-r17 (PMC1431735; doi:10.1186/gb-2006-7-2-r17)
Supplement: Additional File 1 — Genomes used in this study to generate phylogenetic profiles. [file gb-2006-7-2-r17-S1.pdf]

Table 1. Genomes used in this study. The selection was mainly based on Bowers et al. with modifications. We deliberately selected genomes to represent different kingdoms.

| **Taxonomy id** | **Name** | **Lineage** |
|---|---|---|
| 2190 | *Methanocaldococcus jannaschii* | Archaea |
| 2234 | *Archaeoglobus fulgidus* | Archaea |
| 2287 | *Sulfolobus solfataricus* | Archaea |
| 2303 | *Thermoplasma acidophilum* | Archaea |
| 29292 | *Pyrococcus abyssi* | Archaea |
| 50339 | *Thermoplasma volcanium* | Archaea |
| 53953 | *Pyrococcus horikoshii* | Archaea |
| 56636 | *Aeropyrum pernix* | Archaea |
| 64091 | *Halobacterium sp. NRC-1* | Archaea |
| 145262 | *Methanothermobacter thermautotrophicus* | Archaea |
| 139 | *Borrelia burgdorferi* | Bacteria |
| 158 | *Treponema denticola* | Bacteria |
| 160 | *Treponema pallidum* | Bacteria |
| 197 | *Campylobacter jejuni* | Bacteria |
| 287 | *Pseudomonas aeruginosa* | Bacteria |
| 303 | *Pseudomonas putida* | Bacteria |
| 358 | *Agrobacterium tumefaciens* | Bacteria |
| 382 | *Sinorhizobium meliloti* | Bacteria |
| 520 | *Bordetella pertussis* | Bacteria |
| 601 | *Salmonella typhi* | Bacteria |
| 632 | *Yersinia pestis* | Bacteria |
| 666 | *Vibrio cholerae* | Bacteria |
| 747 | *Pasteurella multocida* | Bacteria |
| 782 | *Rickettsia prowazekii* | Bacteria |
| 837 | *Porphyromonas gingivalis* | Bacteria |
| 881 | *Desulfovibrio vulgaris* | Bacteria |
| 956 | *Wolbachia sp.* | Bacteria |
| 1097 | *Chlorobium tepidum* | Bacteria |
| 1148 | *Synechocystis sp. PCC 6803* | Bacteria |
| 1299 | *Deinococcus radiodurans* | Bacteria |
| 1309 | *Streptococcus mutans* | Bacteria |
| 1313 | *Streptococcus pneumoniae* | Bacteria |
| 1314 | *Streptococcus pyogenes* | Bacteria |
| 1351 | *Enterococcus faecalis* | Bacteria |
| 1360 | *Lactococcus lactis subsp. Lactis* | Bacteria |
| 1392 | *Bacillus anthracis* | Bacteria |
| 1423 | *Bacillus subtilis* | Bacteria |
| 1488 | *Clostridium acetobutylicum* | Bacteria |
| 1717 | *Corynebacterium diphtheriae* | Bacteria |
| 1764 | *Mycobacterium avium* | Bacteria |
| 1769 | *Mycobacterium leprae* | Bacteria |
| 1773 | *Mycobacterium tuberculosis* | Bacteria |
| 2097 | *Mycoplasma genitalium* | Bacteria |
| 2104 | *Mycoplasma pneumoniae* | Bacteria |

| | | |
|---|---|---|
| 2107 | *Mycoplasma pulmonis* | Bacteria |
| 2130 | *Ureaplasma urealyticum* | Bacteria |
| 2336 | *Thermotoga maritime* | Bacteria |
| 2371 | *Xylella fastidiosa* | Bacteria |
| 35554 | *Geobacter sulfurreducens* | Bacteria |
| 63363 | *Aquifex aeolicus* | Bacteria |
| 71421 | *Haemophilus influenzae Rd KW20* | Bacteria |
| 83331 | *Mycobacterium tuberculosis CDC1551* | Bacteria |
| 83333 | *Escherichia coli K12* | Bacteria |
| 83334 | *Escherichia coli O157:H7* | Bacteria |
| 83560 | *Chlamydia muridarum* | Bacteria |
| 85962 | *Helicobacter pylori 26695* | Bacteria |
| 85963 | *Helicobacter pylori J99* | Bacteria |
| 86665 | *Bacillus halodurans* | Bacteria |
| 107806 | *Buchnera aphidicola str. APS* | Bacteria |
| 115711 | *Chlamydophila pneumoniae AR39* | Bacteria |
| 115713 | *Chlamydophila pneumoniae CWL029* | Bacteria |
| 122586 | *Neisseria meningitidis MC58* | Bacteria |
| 122587 | *Neisseria meningitidis Z2491* | Bacteria |
| 138677 | *Chlamydophila pneumoniae J138* | Bacteria |
| 155864 | *Escherichia coli O157:H7 EDL933* | Bacteria |
| 158878 | *Staphylococcus aureus subsp. aureus Mu50* | Bacteria |
| 158879 | *Staphylococcus aureus subsp. aureus N315* | Bacteria |
| 3702 | *Arabidopsis thaliana* | Eukaryota |
| 4932 | *Saccharomyces cerevisiae* | Eukaryota |
| 6239 | *Caenorhabditis elegans* | Eukaryota |
| 7227 | *Drosophila melanogaster* | Eukaryota |
