# Supplementary material for: Predicting genes for orphan metabolic activities using phylogenetic profiles
Source: Genome Biol. 2006 Feb 15;7(2):R17. doi: 10.1186/gb-2006-7-2-r17 (PMC1431735; doi:10.1186/gb-2006-7-2-r17)
Supplement: Additional File 2 — The effect of connection specificity adjustment. [file gb-2006-7-2-r17-S2.pdf]

Table 2. The effect of connection specificity adjustment for highly ranked genes (1-50). As shown in the table below, accounting for the specificity of connections between network genes increased predictions, particularly for top ranked genes and genes ranked within 10.

Rank	1	10	20	30	50
No Adjustment	0.20	0.33	0.39	0.41	0.45
Connection Specificity Adjusted	0.23	0.38	0.41	0.43	0.47
Additional Genes Predicted	20	31	10	12	14
