# Supplementary material for: Predicting genes for orphan metabolic activities using phylogenetic profiles
Source: Genome Biol. 2006 Feb 15;7(2):R17. doi: 10.1186/gb-2006-7-2-r17 (PMC1431735; doi:10.1186/gb-2006-7-2-r17)
Supplement: Additional File 3 — Comparison of the performance of the simplex and simulated annealing algorithms. [file gb-2006-7-2-r17-S3.pdf]

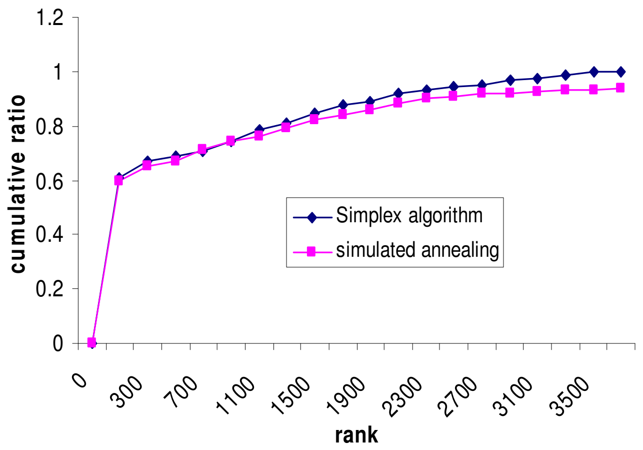

Figure 1. Comparison between the performances of the Simpelx algorithm and the Simulated Annealing algorithm. Cumulative curves are shown. Overall, simulated annealing slightly outperformed simplex, but only on middle- and low-ranked genes.
