# Supplementary material for: Predicting genes for orphan metabolic activities using phylogenetic profiles
Source: Genome Biol. 2006 Feb 15;7(2):R17. doi: 10.1186/gb-2006-7-2-r17 (PMC1431735; doi:10.1186/gb-2006-7-2-r17)
Supplement: Additional File 4 — Comparison of the predictions based on Equations 2 and 3. [file gb-2006-7-2-r17-S4.pdf]

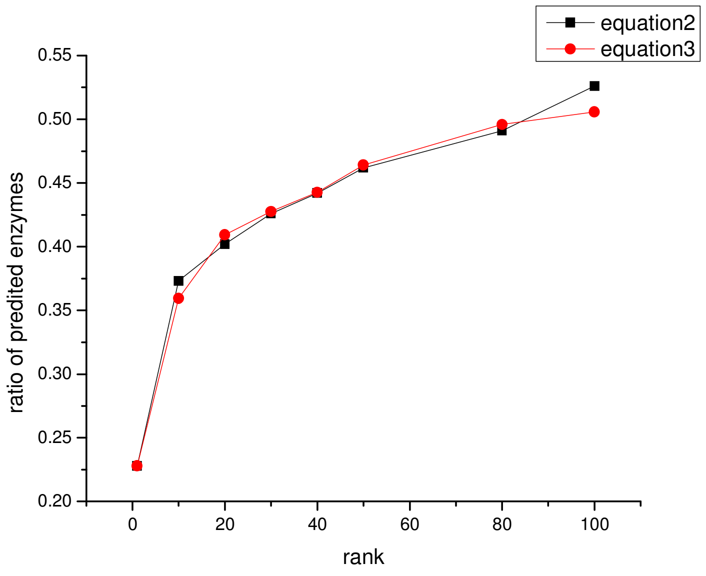

Figure 2. Prediction based on cost functions Equation 2 or 3 (see text). Only highly ranked genes were reported (i.e., rank 1-100). Very similar performances were observed for Equation 2 and 3. Results in the paper were based on Equation 2.
