# Supplementary material for: Predicting genes for orphan metabolic activities using phylogenetic profiles
Source: Genome Biol. 2006 Feb 15;7(2):R17. doi: 10.1186/gb-2006-7-2-r17 (PMC1431735; doi:10.1186/gb-2006-7-2-r17)
Supplement: Additional File 5 — Ten-fold cross-validation of the algorithm. [file gb-2006-7-2-r17-S5.pdf]

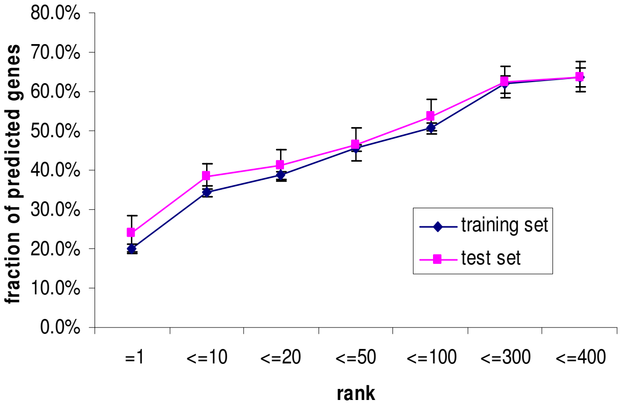

Figure 3. 10-fold cross-validation of the predictive algorithm. Error bars represent the variations in terms of prediction over the sample sets. Similar results were obtained for the training sets and test sets, suggesting overfitting is not a problem of the method.
