# Supplementary material for: Predicting genes for orphan metabolic activities using phylogenetic profiles
Source: Genome Biol. 2006 Feb 15;7(2):R17. doi: 10.1186/gb-2006-7-2-r17 (PMC1431735; doi:10.1186/gb-2006-7-2-r17)
Supplement: Additional File 6 — Comparison of the predictions based on all yeast non-metabolic genes as the candidate gene set, all hypothetical genes or a randomly selected subset of yeast non-metabolic genes. [file gb-2006-7-2-r17-S6.pdf]

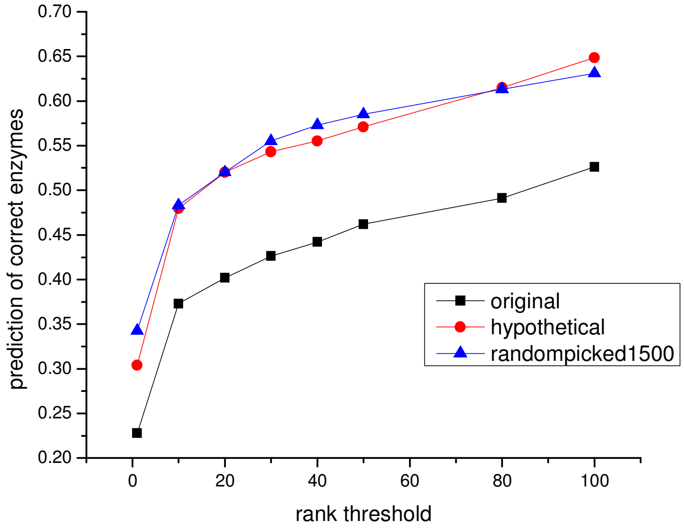

Figure 4. Comparison of the predictions based on three different sets of candidate genes: all 6094 candidate genes, 1514 hypothetical genes and 1514 randomly selected known non-metabolic genes. There is about 40-50% increase in terms of predictive power when only the hypothetical genes were used. But the increment is mainly due to the smaller number of candidate set, as we observed almost identical performance in the randomly selected 1514 gene set.
