# Supplementary material for: Predicting genes for orphan metabolic activities using phylogenetic profiles
Source: Genome Biol. 2006 Feb 15;7(2):R17. doi: 10.1186/gb-2006-7-2-r17 (PMC1431735; doi:10.1186/gb-2006-7-2-r17)
Supplement: Additional File 7 — Context-based association as a function of metabolic network distance in E. coli. [file gb-2006-7-2-r17-S7.pdf]

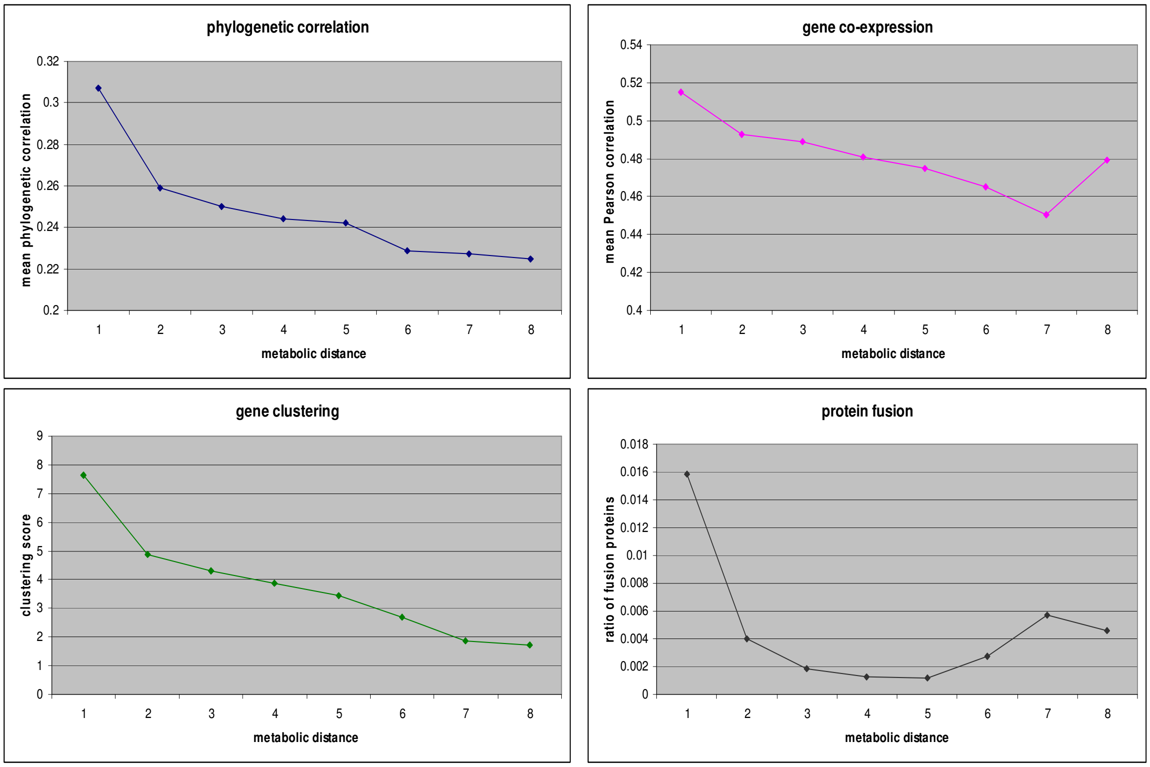

Fig 5. Context-based measurements versus the network distance in the E. coli metabolic network. Figure shows (a). Gene phylogenetic Pearson correlation. (b). Gene co-expression Pearson correlation. (c). Gene clustering score and (d) Protein fusion events. In general, context-based scores decrease monotonically as the metabolic distance between two genes increases. Gene clustering scores were calculated as log10(clustering probability). The larger the score, the more likely two genes cluster. Protein fusion events were calculated as the number of pairs of genes fused divided by total number of gene pairs at a certain metabolic distance.
