# Supplementary material for: Predicting genes for orphan metabolic activities using phylogenetic profiles
Source: Genome Biol. 2006 Feb 15;7(2):R17. doi: 10.1186/gb-2006-7-2-r17 (PMC1431735; doi:10.1186/gb-2006-7-2-r17)
Supplement: Additional File 8 — Comparison of predictions based on normalized gene phylogenetic profiles, mutual information, and the method reported in the paper. [file gb-2006-7-2-r17-S8.pdf]

Section 1. Prediction based upon normalized phylogenetic profiles and mutual information.

We also tested the effect of phylogenetic profile normalization in our prediction. The method proposed by Date et al.[42] is supposed to generate real value phylogenetic profiles to account for the divergence between protein sequences (see methods). However, we did not see significant improvement using normalized phylogenetic profiles. Upon convergence, the algorithm predicts 16%, 32%, 47% and 64% metabolic genes to be top ranked, within top 10, within top 50 and within top 300.

We also tested the system performance while using mutual information as the input context-based data. We found that using of MI created from the binary (E-value threshold = 0.001) phylogenetic profile provided slightly worse performance while MI created using normalized phylogenetic profiles displayed comparable predictions, with the former predicting 9%, 16%, 28.3% and 56.6% and the latter predicting 20.0%, 35.8%, 43.3% and 52.5% of correct metabolic genes to be top ranked, within top 10, within top 50 and within top 300 among all candidate genes, respectively. Overall, these experiments suggested that use of binary phylogenetic profiles and correlations based on binary phylogenetic profiles is probably sufficient for our prediction algorithm. But since the procedure of generating binary phylogenetic profiles is much more straightforward than other type of data, in practice, we mainly used the binary phylogenetic profiles in prediction.

[42] Date, S. V. & Marcotte, E. M. (2003) *Nat Biotechnol* **21,** 1055-62.
